# Exosomes Derived from Baicalin-Pretreated Mesenchymal Stem Cells Alleviate Hepatocyte Ferroptosis after Acute Liver Injury via the Keap1-NRF2 Pathway

**DOI:** 10.1155/2022/8287227

**Published:** 2022-07-21

**Authors:** Shuxian Zhao, Mengxin Huang, Lili Yan, Hao Zhang, Chenchen Shi, Jing Liu, Shousong Zhao, Hongbo Liu, Baogui Wang

**Affiliations:** ^1^Department of Infectious Disease, The Affiliated Fuyang Hospital of Bengbu Medical College, Fuyang People's Hospital, Fuyang, 236000 Anhui, China; ^2^Department of Infectious Disease, The First Affiliated Hospital of Bengbu Medical College, Bengbu, 233400 Anhui, China; ^3^Department of Emergency Medicine, Fuyang People's Hospital, Fuyang, 236000 Anhui, China

## Abstract

Acute liver injury (ALI) is characterized as a severe metabolic dysfunction caused by extensive damage to liver cells. Ferroptosis is a type of cell death dependent on iron and oxidative stress, which differs from classical cell death, such as apoptosis and necrosis. Ferroptosis has unique morphological features, which mainly include mitochondrial dissolution and mitochondrial outline reduction. Furthermore, the intracellular accumulation of lipid peroxides directly affects the occurrence of ferroptosis. Baicalin, the main compound isolated from *Scutellaria baicalensis*, has anti-inflammatory and antioxidative effects. Recently, exosomes derived from preconditioned mesenchymal stem cells (MSCs) have shown great potential in the treatment of various diseases including ALI. This study investigates the ability of exosomes derived from baicalin-pretreated MSCs (Ba-Exo) to promote liver function recovery in mice with ALI compared with those without pretreatment. Through in vivo and in vitro experiments, this study demonstrates for the first time that Ba-Exo greatly attenuates D-galactosamine and lipopolysaccharide (D-GaIN/LPS)-induced liver damage and inhibits reactive oxygen species (ROS) production and lipid peroxide-induced ferroptosis. Moreover, P62 was significantly upregulated in Ba-Exo, whereas its downregulation in Ba-Exo counteracted the beneficial effect of Ba-Exo. P62 regulates hepatocyte ferroptosis by activating the Keap1-NRF2 pathway. The beneficial effect of Ba-Exo in inhibiting ferroptosis was also attenuated after the NRF2 pathway was inhibited. Therefore, baicalin pretreatment is an effective and promising approach to optimize the therapeutic efficacy of MSC-derived exosomes in ALI.

## 1. Introduction

Acute liver injury (ALI) is a severe metabolic dysfunction, which is attributed to extensive liver cell damage [1]. ALI is commonly seen in various serious liver diseases. Moreover, severe liver damage is caused by various extrahepatic factors.

Ferroptosis is a recently discovered, novel type of iron-dependent, lipid peroxide accumulation-induced, nonapoptotic cell death, which has different morphological and biochemical characteristics from other cell deaths [2]. Recent studies have found that the pathogenesis of ALI is related to ferroptosis. For example, in a liver ischemia-reperfusion model, ferroptosis-related inhibitors improved mouse mortality [3]. Several studies have also shown that ferroptosis inhibition is a promising research strategy for liver injury disease treatment [4]. Wu et al. demonstrated that fibroblast growth factor 21 alleviates iron overload-induced liver injury and fibrosis by inhibiting ferroptosis [5]. Furthermore, protecting the mitochondria by inhibiting VDAC1 oligomerization could alleviate ferroptosis in hepatocytes by restoring ceramide and cardiolipin content in ALI [6].

Mesenchymal stem cells (MSCs)-based therapy has emerged as a promising strategy for the treatment of liver diseases via tissue repair and immunomodulation [7]. However, the direct transplantation of stem cells into the target tissue poses certain risks, such as cell dedifferentiation risk, poor survival, and tumor formation [8]. Additionally, related ectopic tissue formation, infusion toxicity caused by cell retention in pulmonary microvessels, cell rejection, or unnecessary transplantation further limit the clinical application of direct stem cell transplantation in the treatment of ALI.

Studies report that transplanted stem cells mainly exert therapeutic effects through paracrine mechanisms, with exosomes playing an important role [9]. MSCs-derived exosomes (MSCs-Exo) have been widely reported to have therapeutic benefits in various in vitro functional assays and related preclinical disease models [10–12]. Our previous study also showed that MSCs-Exo can alleviate hepatocyte apoptosis [13]. Notably, MSCs-Exo have been successfully reproduced, with their therapeutic effects confirmed in relevant in vitro and in vivo disease models.

Exosomes are membrane-derived nanoscale vesicles (30-150 nm) that are released from different cell types under normal or pathological conditions and affect recipient cell activity by inducing signaling pathways [14]. Exosomes contain not only cellular proteins and lipids but also host cell mRNAs and micro-RNAs (miRNAs) [15]. The use of MSCs-Exo has produced beneficial effects in various animal models of liver diseases, including drug-induced ALI, liver fibrosis, and hepatocellular carcinoma [12, 16, 17]. Exosomes have various advantages over their corresponding MSCs: they are smaller and simpler than cells and, hence, easier to produce and store, thereby potentially avoiding few MSC-related regulatory issues. Therefore, MSCs-Exo could be ideal therapeutic tools for liver diseases.

Preconditioning using cytokines, drugs, hypoxic conditions, or physical factors can improve the transplantation efficacy, thereby improving the biological function of MSCs and enhancing the paracrine effect [18]. Baicalin is a flavonoid glycoside that is extracted from the root of *Scutellaria baicalensis*. Baicalin has been reported to possess various pharmacological effects, including anti-inflammatory, antibacterial, antifibrotic, antioxidative, and anticancerous effects [19–21]. Additionally, baicalin protects against ALI by reducing liver inflammation [22, 23]. Furthermore, Shi et al. showed that baicalin attenuated Acetaminophen (APAP)-induced ALI by inducing NRF2 activation and NLRP3 inflammasome activation [24]. However, the benefits of exosomes derived from baicalin-pretreated mesenchymal stem cells (Ba-Exo) in liver function recovery remain unclear; moreover, it is yet to be determined whether this benefit could be attributed to the genetic material in the exosomes.

This study investigates the protective effect of Ba-Exo on liver function by establishing a D-galactosamine and lipopolysaccharide (D-GaIN/LPS)-induced ALI model and further explores its protective mechanism on hepatocyte ferroptosis. Additionally, the potential efficacy of inhibiting ferroptosis in hepatocytes and alleviating liver injury by Ba-Exo has been shown. Notably, Ba-Exo exerts a protective effect on liver function by inducing P62 to activate the Keap1-NRF2 pathway. Therefore, this study elucidates the potential mechanism of action of Ba-Exo, providing an effective and promising approach for the treatment of ALI.

## 2. Material and Method

### 2.1. ALI Model Establishment and Exosomes Injection

C57BL/6 mice at 6-8 weeks were intraperitoneally injected with D-GalN/LPS (1772-03-8/L2880, Sigma-Aldrich, USA) at a dose of 700 mg/kg and 10 *μ*g/kg, respectively. The constructed D-GaIN/LPS-induced ALI model mice were named the model group, and the normal mice injected with phosphate-buffered saline (PBS) were named the blank group. After 1 h of LPS/D-GalN treatment, Exo and Ba-Exo (150 *μ*g/mice) were injected into the tail vein of the mice in the Exo and Ba-Exo groups, respectively. Mice were sacrificed via anesthesia overdose 12 h after the intervention. Half of the liver tissue was fixed in paraformaldehyde, while the other half was frozen at 80°C. Peripheral blood serum was stored at -80°C.

### 2.2. Serum Alanine Aminotransferase (ALT) and Aspartate Aminotransferase (AST) Level Analysis

The levels of ALT and AST in mouse serum were used to determine liver function. The blood samples were stored at room temperature for 2 h, and the serum was collected after centrifugation for 15 min at 840 × g. The levels of ALT and AST in serum were detected following the manufacturer's instructions (1167/8674, MEIMIAN).

### 2.3. Enzyme-Linked Immunosorbent Assay (ELISA) Analysis

TNF-a, IL-6, and MCP-1 levels in liver tissue homogenates were measured using commercially available ELISA kits (EK282/EK206/EK287, LiankeBio), following the manufacturer's instructions.

### 2.4. Dihydroethidium (DHE) Staining

Reactive oxygen species (ROS) levels were assessed using a fluorescent staining method with the ROS probe DHE. Liver tissue sections were incubated with 10 *μ*M DHE for 30 min to detect intracellular ROS generation. Signals in liver samples were quantified using fluorescence microscopy and NIH ImageJ software. After the DHE staining was completed, we randomly selected three slices from each group and five different fields of view per slice.

### 2.5. Hepatocyte Extraction

Hepatocytes were extracted based on a previous report [13]. Briefly, mouse livers were extracted, and the liver capsule was torn off to obtain thin tissue sections. After cold digestion with 0.25% trypsin (Gibco) at 4°C for 10-12 h, the cells were ground using a 200-mesh sieve to obtain a single-cell suspension, which contained PBS buffer. The suspension was centrifuged thrice at 1000 r/min for 5 min, discarding the supernatant each time. Hepatocytes were pelleted and cultured in Dulbecco's modified Eagle's medium (DMEM) (Thermo Fisher Scientific, MA, USA) containing 10% fetal bovine serum. The hepatocytes were cultured in a CO_2_ incubator at 37°C for 12 h, and the cells adhering to the wall for used for further experiments.

### 2.6. Fe^2+^, Malondialdehyde (MDA), Glutathione (GSH), and ROS Detection

Fe^2+^ content, MDA, and GSH expression in liver tissue homogenates or cells were detected different using kits (ab83366/ab118970/ab138881, Abcam), following the manufacturer's protocol. The ROS Assay Kit (S0033S, Beyotime Biotechnology, China) was used to quantify ROS production in cells using a fluorescence microplate reader. Analyses were performed using flow cytometry (Beckman).

### 2.7. Exosome Isolation and Identification

Exosomes were isolated from MSC supernatants using ultracentrifugation based on a previous report [13]. Briefly, low-speed centrifugation at 300 g for 10 min was performed to remove cellular components. Low-speed centrifugation and centrifugation at 2000 g for 10 min was further performed to remove dead cells. Centrifugation at 10,000 g for 70 min was also performed to eliminate cell debris. High-speed centrifugation at 120,000 g for 60 min was performed to obtain exosomes as pellets. The exosomes were resuspended in an appropriate amount of PBS to eliminate contaminating proteins and subsequently centrifuged at 120,000 g for 60 min. The pellet was collected and resuspended in PBS. The protein concentration was determined using the bicinchoninic acid assay, and the samples were stored at -80°C. Exosomes were analyzed using western blot, nanoparticle tracking analysis (NTA) and transmission electron microscopy (TEM). Hepatocytes were seeded in confocal dishes and incubated overnight. Exosomes were fluorescently labeled with PKH26 (MINI26, Sigma) and incubated with hepatocytes for 24 h. The uptake of exosomes was observed under a laser confocal microscope (LEICA).

### 2.8. Western Blot

Proteins from cells and tissues were extracted using a whole protein extraction kit (KGP2100, KeyGEN BioTECH) following the manufacturer's protocol. Ten microliters of each sample were loaded on a 15% sodium dodecyl sulfate-polyacrylamide gel and separated using electrophoresis. The gel was then transferred to a nitrocellulose membrane. The membrane was blocked with 5% nonfat milk and then dissected according to the molecular weight indicated by the prestained markers. Primary antibody was added to the membranes and incubated overnight at 4°C on a shaker. Membranes were washed thrice with Tris-buffered saline with tween (TBST) and incubated with horseradish peroxidase-conjugated secondary antibody (1 : 5000) for 1 h at room temperature. After rinsing thrice with TBST, the membrane was developed using electrochemiluminescence, exposed, and analyzed using ImageJ software. The primary antibodies are as follows: anti-CD9 (20597-1-AP, Proteintech, 1 : 3000), anti-CD63 (25682-1-AP, Proteintech, 1 : 1000), anti-CD81 (ab79559, Abcam, 1 : 1000), anti-TSG101 (ab125011, Abcam,1 : 5000), anti-5-LOX (ab169755, Abcam,1 : 1000), anti-SLC7A11 (26864-1-AP, Proteintech,1 : 1000), anti-GPX4 (ab125066, Abcam,1 : 5000), anti-IL-6 (21865-1-AP, Proteintech,1 : 1000), anti-MCP-1 (66272-1-Ig, Proteintech, 1 : 2000), anti-TNF-*α* (ab183218, Abcam, 1 : 1000), anti-P62 (18420-1-AP, Proteintech, 1 : 5000), anti-Keap1 (8047S, Cell Signaling Technology, 1 : 1000), anti-NRF2 (16396-1-AP, Proteintech, 1 : 1000), and anti-GAPDH (60004-1-Ig, Proteintech, 1 : 10000).

### 2.9. Cell Transfection

The lentivirus of shP62 was constructed by Hanheng Biotechnology. The scrambled lentiviral construct served as a negative control. Transfections were performed using Lipofectamine 3000 reagent (L3000015, Thermo Fisher Scientific), following the manufacturer's instructions.

### 2.10. Immunofluorescence

Cells were fixed, disrupted, and blocked with anti-NRF2 (16396-1-AP, Proteintech, 1 : 200) and incubated overnight at 4°C. Alexa Flour 594-conjugated goat secondary antibody (A12922, Thermo Fisher Science) was added, and the cells were incubated for 1 hr at room temperature. Nuclei were stained using 4′,6-diamidino-2-phenylindole (DAPI) (C1006, Beyotime Biotechnology, China), and fluorescence images were acquired using a fluorescence microscope. Five visual fields were randomly selected, and the average was obtained.

### 2.11. Reverse Transcription-Polymerase Chain Reaction (RT-PCR)

Total RNA was extracted from the liver using TRIzol reagent (15596026, Invitrogen). cDNA was synthesized, and RT-PCR was performed. The relative expression of the target gene was normalized against *β*-actin and analyzed using the 2^-*ΔΔ*Ct^ method.

### 2.12. Statistical Analyses

Statistical analyses were performed using GraphPad Prism 5.0 software. Data are presented as mean ± standard deviation. Statistical significance among multiple groups was assessed using a one-way or two-way analysis of variance followed by Dunnett's post hoc test. *P* < 0.05 was considered statistically significant.

## 3. Results

### 3.1. Identification and Uptake of Exo and Ba-Exo

**Figure 1:**
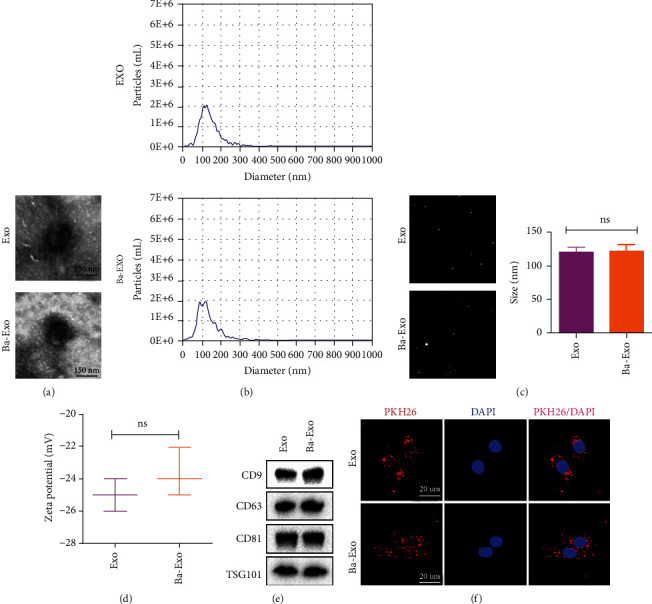
Identification of Exo and Ba-Exo and the uptake of Ba-Exo by hepatocytes: (a) the morphology of Exo and Ba-Exo was detected using transmission electron microscopy; (b, c) the particle size of Exo and Ba-Exo was analyzed using nanoparticle tracking analysis; (d) western blot detection of exosome surface markers, such as CD9, CD63, CD81, and TSG101; (e) the zeta potential of Exo and Ba-Exo; (f) the uptake of exosomes labeled with red fluorescent dye PKH26 by hepatocytes.

TEM revealed that Exo and Ba-Exo have similar cup-like or spherical morphologies (Figure 1(a)). NTA confirmed that the particle size distribution of Exo and Ba-Exo nanoparticles was between 30 and 150 nm (Figures 1(b) and 1(c)), with no significant difference in zeta potential between them (Figure 1(d)). Western blot analysis revealed the presence of exosome surface markers CD9, CD63, CD81, and TSG101 in both Exo and Ba-Exo (Figure 1(e)). Additionally, the confocal microscopy images of Exo and Ba-Exo labeled with PKH26 dye and cocultured with hepatocytes for 24 h showed the presence of PKH26-labeled exosomes in the cytoplasm, confirming the uptake of exosomes by hepatocytes (Figure 1(f)).

### 3.2. Ba-Exo Reduces the Expression of ALT, AST, and Inflammatory Factors after ALI

**Figure 2:**
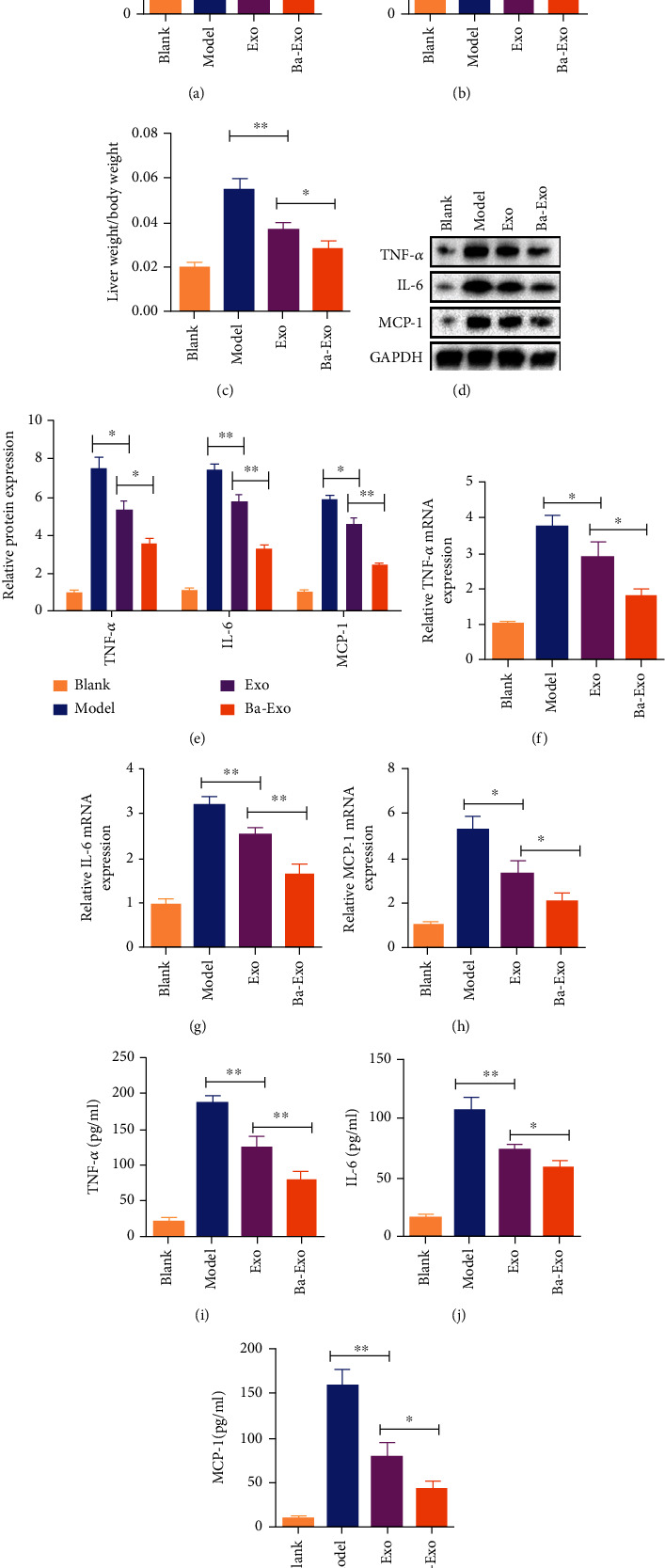
Ba-Exo reduces the expression of alanine aminotransferase (ALT), aspartate aminotransferase (AST), and inflammatory factors after acute liver injury. (a, b) Changes in serum ALT and ASL levels; (c) liver weight to body weight ratio; (d, e) the western blot analysis of TNF-*α*, IL-6, and MCP-1 expressions in liver tissue; (f–h) quantitative reverse transcription-polymerase chain reaction analysis of TNF-*α* mRNA, IL-6 mRNA, and MCP-1 mRNA expression in liver tissue; (i–k) enzyme-linked immunosorbent assay detects TNF-*α*, IL-6, and MCP-1 expression in liver tissue.

Exo have been previously reported to alleviate hepatocyte damage after ALI in mice [13, 25]. This study investigated whether Ba-Exo has a greater beneficial effect than Exo. The levels of ALT and AST in the plasma of mice with ALI were evaluated, wherein the concentrations of ALT and AST in the serum were significantly increased (Figures 2(a) and 2(b)). Exo and Ba-Exo treatment effectively inhibited ALT and AST concentrations in the plasma of liver-injured mice. Notably, Ba-Exo exhibited a more significant inhibitory effect than Exo (Figures 2(a) and 2(b)). Additionally, this study quantitatively analyzed liver enlargement by weighing mice body weight and mouse liver weight, thereby calculating the liver weight/body weight ratio. Compared with the blank group, the liver weight/body weight ratio of the mice in the model group was significantly increased (Figure 2(c)). Compared with the model group, the hepatomegaly of the Exo and Ba-Exo groups was relieved. However, the liver weight/body weight ratio of the Ba-Exo group was further reduced compared with the Exo group (Figure 2(c)). Therefore, the administration of Ba-Exo has the potential to more effectively attenuate D-GaIN/LPS-induced ALI.

The expression levels of inflammatory factors TNF-a, IL-6, and MCP-1 in mice were determined using western blot, RT-PCR, and ELISA. Western blot showed that TNF-a, IL-6, and MCP-1 expressions in the liver tissue homogenate of the model group were significantly increased, whereas Exo and Ba-Exo significantly inhibited TNF-a, IL-6, and MCP-1 expression (Figures 2(d) and 2(e)). Additionally, the levels of TNF-a, IL-6, and MCP-1 in the liver tissue homogenate were further decreased in the Ba-Exo group compared with the Exo group (Figures 2(d) and 2(e)). Furthermore, these results were validated via RT-PCR and ELISA analyses (Figures 2(f)–2(k)). Therefore, the use of Ba-Exo is more effective in attenuating the release of inflammatory factors following ALI than Exo alone.

### 3.3. Ba-Exo Attenuates Ferroptosis in Hepatocytes after ALI

**Figure 3:**
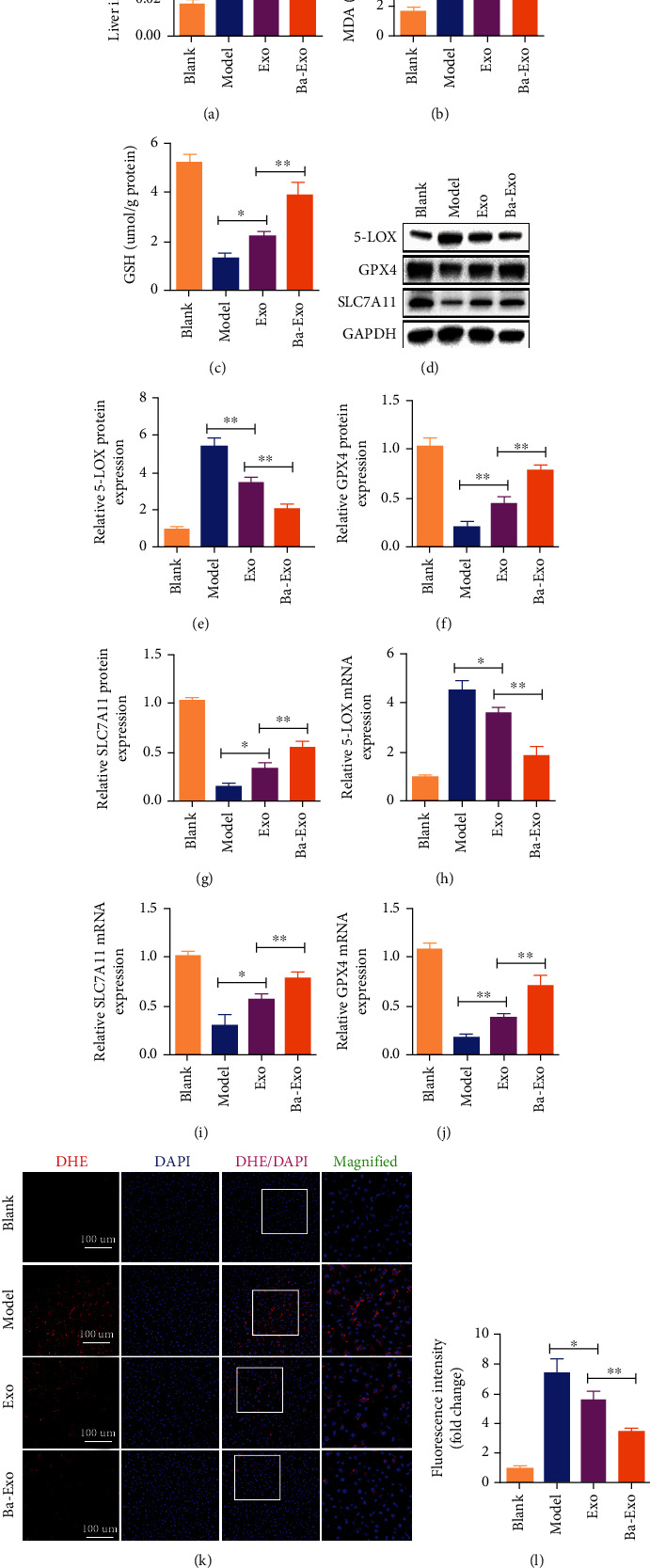
Ba-Exo alleviates ferroptosis in hepatocytes after acute liver injury. (a) Changes in iron content in liver tissue; (b, c) glutathione (GSH) and malondialdehyde (MDA) measurements; (d–g) western blot detection of the changes in ferroptosis protein markers; (h–j) quantitative reverse transcription-polymerase chain reaction analysis of the mRNA content of ferroptosis markers; (k, l) dihydroethidium staining and fluorescence quantitative analysis of liver tissue.

As shown in Figure 3(a), iron concentration in the liver tissue of mice was significantly increased 12 h after D-GaIN/LPS administration but decreased after Exo and Ba-Exo treatment. However, the liver tissue iron concentration was lower after Ba-Exo treatment than that with Exo treatment. Additionally, lipid peroxide measurement showed that MDA content in the liver tissue of the mice in the model group was significantly higher than that in the blank group (Figure 3(b)). The Ba-Exo intervention significantly reduced MDA levels in the model group (Figure 3(b)). Moreover, compared with the model group, the GSH content in the liver tissue of the Ba-Exo group was significantly improved (Figure 3(c)). Additionally, the detection of changes in ferroptosis markers (5-LOX, GPX4, and SLC7A11) using western blot and RT-PCR revealed abnormal changes in ferroptosis markers. Ba-Exo treatment inhibited the decrease of GPX4 and SLC7A11 proteins and the increase of 5-LOX protein (Figures 3(d)–3(g)). Furthermore, RT-PCR detection confirmed the western blot results (Figure 3(h)–3(j)). Finally, the evaluation of ROS production in liver tissue using DHE staining revealed that the ROS levels in the model group were significantly increased, whereas ROS fluorescence intensity decreased after Exo and Ba-Exo treatments, with Ba-Exo treatment showing more obvious effects (Figure 3(k) and 3(l)). Therefore, Ba-Exo has the potential to effectively inhibit lipid peroxidation-induced ferroptosis in D-GaIN/LPS-induced ALI.

### 3.4. Ba-Exo Inhibits Erastin-Induced Ferroptosis in Hepatocytes

**Figure 4:**
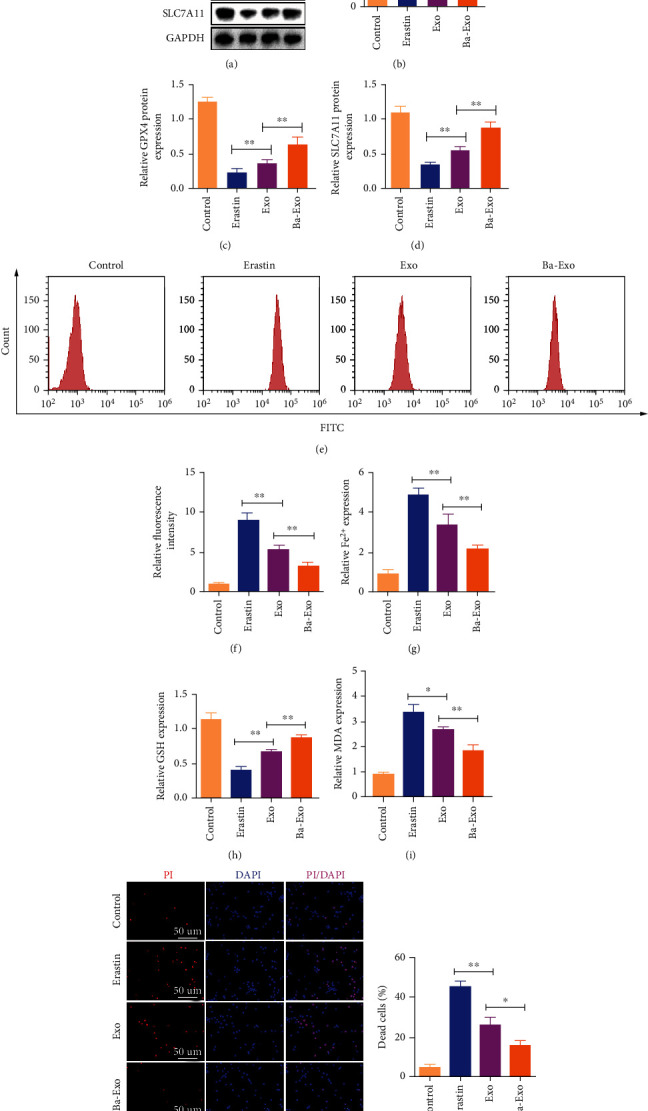
Ba-Exo inhibits Erastin-induced ferroptosis in hepatocytes. (a–d) Western blot analysis of ferroptosis protein marker expressions; (e, f) flow cytometry detection of reactive oxygen species levels; (g) Fe^2+^ measurement; (h, i) glutathione (GSH) and malondialdehyde (MDA) measurements; (j, k) propidium iodide staining to detect cell death.

To further verify the inhibitory effect of Ba-Exo on ferroptosis after ALI, hepatocytes were pretreated with Ba-Exo (150ug/ml) in vitro, and the ferroptosis inducer Erastin (10 *μ*M, 6 h) was used to treat the hepatocytes. Western blot assessed the changes in ferroptosis markers, revealing that Ba-Exo treatment inhibited the decrease of GPX4 and SLC7A11 proteins and the increase of 5-LOX protein, confirming the in vivo results (Figures 4(a)–4(d)). The accumulation of lipid ROS in cells is a typical feature of ferroptosis and a key factor in the induction of ferroptosis. The effect of Ba-Exo on ROS levels in hepatocytes was assessed using flow cytometry, which detected the changes in ROS levels in cells. Ba-Exo inhibited Erastin-induced ROS levels more effectively than Exo (Figures 4(e) and 4(f)). Additionally, Ba-Exo pretreatment more effectively reduced the increased intracellular Fe^2+^ levels (Figure 4(g)) than Exo. Similarly, oxidative stress indicator analysis showed that Ba-Exo was more effective in increasing intracellular GSH levels and reducing MDA content than Exo (Figures 4(h)–4(i)). Furthermore, propidium iodide (PI) staining showed that Ba-Exo pretreatment significantly inhibited Erastin-induced cell death compared with Exo (Figures 4(j) and 4(k)). Therefore, Ba-Exo has a better ability to effectively inhibit Erastin-induced ferroptosis and exert a protective effect than Exo.

### 3.5. Ba-Exo Attenuates Hepatocyte Ferroptosis Induced by ALI via P62 In Vivo

**Figure 5:**
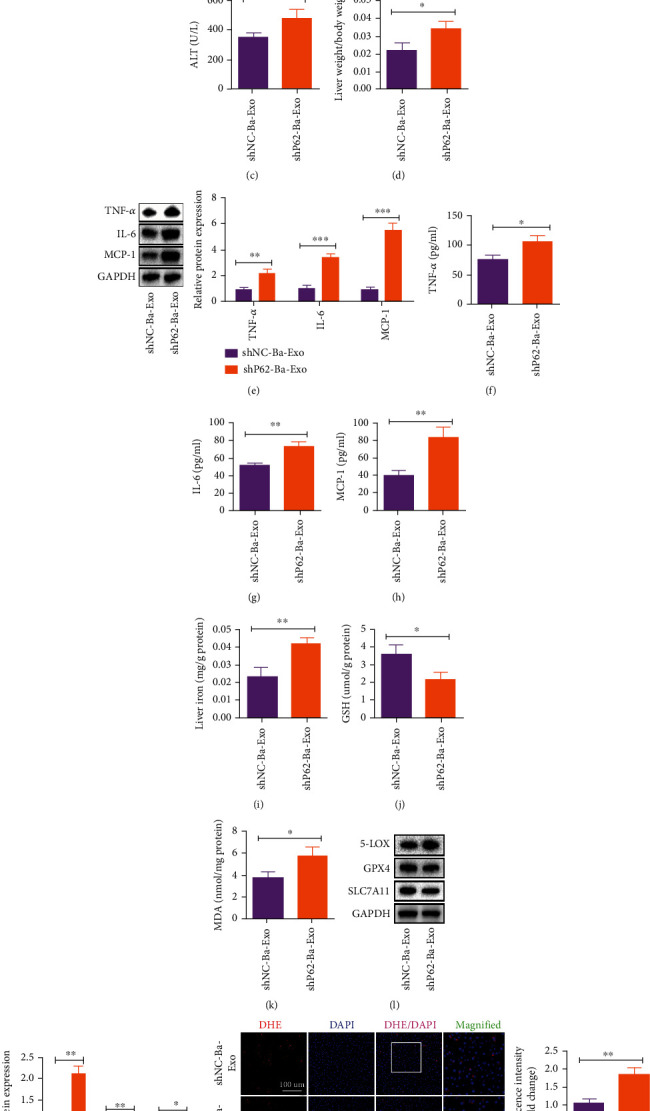
Ba-Exo attenuates hepatocyte ferroptosis induced by acute liver injury via P62 in vivo. (a) Western blot detection of P62 protein expression in exosomes; (b, c) changes in serum alanine aminotransferase (ALT) and aspartate aminotransferase (AST) levels; (d) the ratio of liver weight to body weight; (e) western blot detection of TNF-*α*, IL-6, and MCP-1 expressions in liver tissue; (f–h) enzyme-linked immunosorbent assay detection of TNF-*α*, IL-6, and MCP-1 expression in liver tissue; (i) changes in iron content in liver tissue; (j, k) glutathione (GSH) and malondialdehyde (MDA) measurements; (l, m) western blot detection of the changes in ferroptosis protein markers; (n) dihydroethidium staining and quantitative fluorescence analysis of liver tissue.

Ba-Exo was found to contain more P62 proteins than Exo. Therefore, the inhibitory effect of Ba-Exo on ferroptosis was speculated to be carried out via the P62 protein. To test this hypothesis, P62 was knocked down in MSCs using a lentivirus-based approach. Exosomes were isolated and cocultured with hepatocytes. The expression of P62 was significantly decreased in shP62-Ba-Exo compared with shNC-Ba-Exo (Figure 5(a)). To further investigate the role of P62 in Ba-Exo-mediated ferroptosis inhibition and liver function protection, shNC-Ba-Exo and shP62-Ba-Exo were administered in mice with ALI. The analysis of ALT, AST, and liver weight/body weight ratio showed that shP62-Ba-Exo treatment resulted in the poor recovery of liver function than shNC-Ba-Exo treatment (Figures 5(b)–5(d)). The role of P62 in liver tissue inflammatory factors TNF-a, IL-6, and MCP-1 was further studied using western blot, wherein the inhibitory effects of shNC-Ba-Exo on TNF-a, IL-6, and MCP-1 were reversed after treatment with shP62-Ba-Exo (Figure 5(e)). ELISA results confirmed these observations (Figures 5(f)–5(h)). Furthermore, shP62-Ba-Exo treatment inhibited liver tissue iron concentration reduction, GSH reduction, and MDA increase compared with shNC-Ba-Exo treatment (Figures 5(i)–5(k)). Western blot detection of ferroptosis markers and DHE staining further confirmed that shP62-Ba-Exo treatment attenuated the inhibitory effect of shNC-Ba-Exo on ferroptosis (Figures 5(l)–5(n)). Therefore, the ability of Ba-Exo to inhibit ferroptosis and attenuate liver injury in mice is dependent on P62.

### 3.6. P62 Inhibits Erastin-Induced Ferroptosis in Hepatocytes

**Figure 6:**
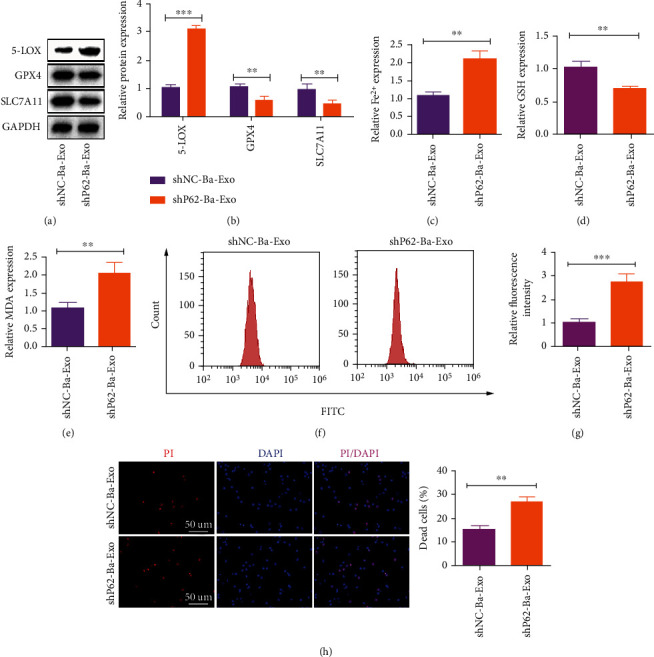
Ba-Exo delivers P62 in vitro to inhibit Erastin-induced ferroptosis in hepatocytes. (a, b) Western blot detection of ferroptosis protein marker levels; (c) Fe^2+^ measurement; (d, e) glutathione (GSH) and malondialdehyde (MDA) measurements; (f, g) Detection of reactive oxygen species levels using flow cytometry; (h) propidium iodide staining to detect cell death.

Next, in vitro cultured hepatocytes were treated with shNC-Ba-Exo and shP62-Ba-Exo to explore the role of P62 in the in vitro hepatocyte response to Erastin-induced ferroptosis. Western blot revealed that shP62-Ba-Exo treatment significantly reversed the changes in ferroptosis markers compared with shNC-Ba-Exo (Figures 6(a) and 6(b)). Furthermore, shP62-Ba-Exo treatment suppressed the cellular Fe^2+^ concentration decrease, GSH decrease, and MDA increase compared with shNC-Ba-Exo treatment (Figures 6(c)–6(e)). Flow cytometry and PI staining revealed that shP62-Ba-Exo treatment reversed ROS level reduction and cell survival caused by shNC-Ba-Exos (Figures 6(f) and 6(h)). These in vitro results highlight the importance of P62 in Ba-Exo in regulating hepatocyte ferroptosis.

### 3.7. Ba-Exo Inhibits Erastin-Induced Ferroptosis in Hepatocytes by Activating the Keap1-NRF2 Pathway

**Figure 7:**
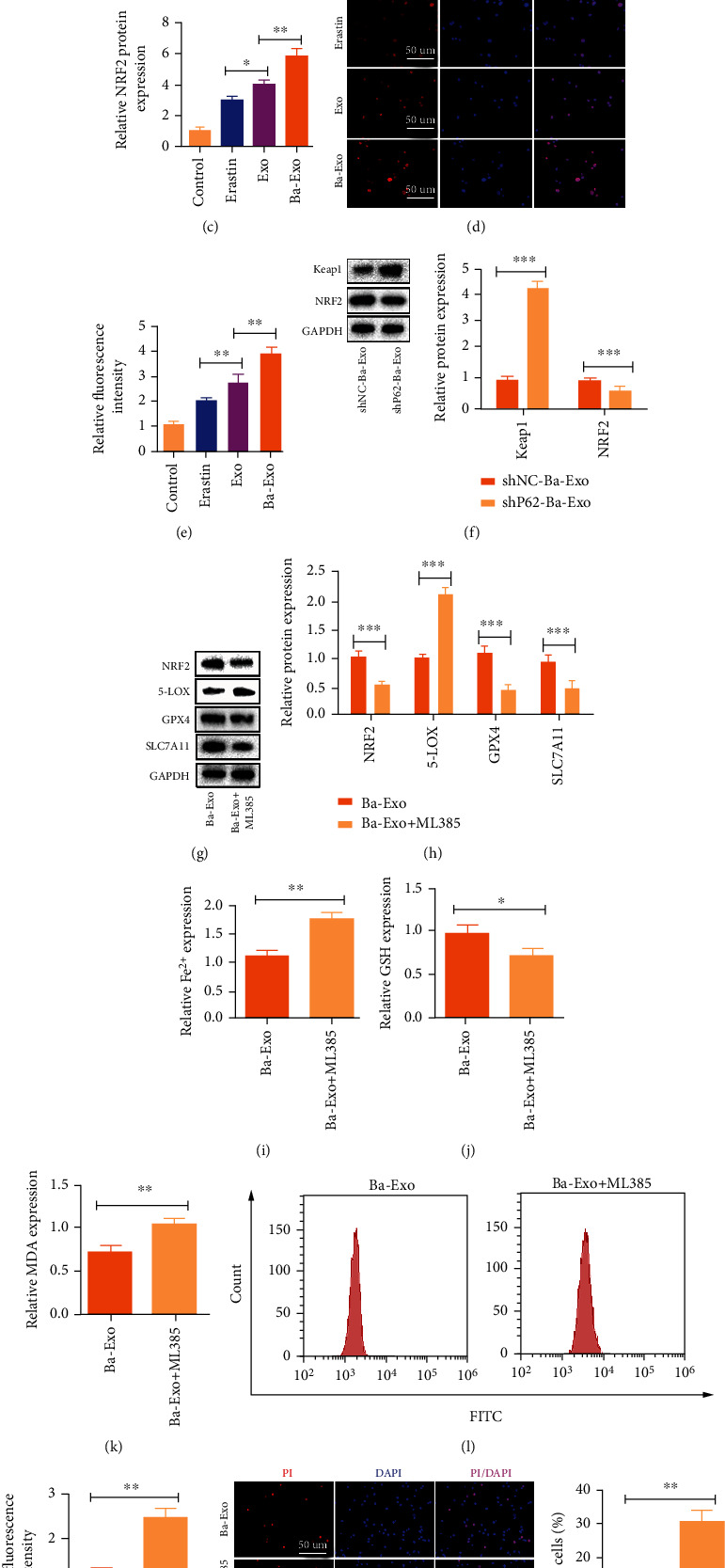
Ba-Exo inhibits Erastin-induced ferroptosis in hepatocytes by activating the Keap1-NRF2 pathway. (a–c) Western blot analysis of Keap1 and NRF2 protein expressions; (d, e) immunofluorescence staining to detect NRF2 expression level; (f) western blot analysis of NRF2 and Keap1 protein expressions; (g, h) western blot analysis of Keap1, NRF2, 5-LOX, GPX4, and SLC7A11 protein expressions; (i–k) measurement of Fe^2+^, malondialdehyde (MDA), and glutathione (GSH); (l, m) detection of reactive oxygen species levels using flow cytometry; (n) propidium iodide staining to detect cell death.

The P62-Keap1-NRF2 signaling pathway plays an important role in protecting hepatocytes against iron toxicity by upregulating multiple genes involved in iron and ROS metabolisms [26]. Therefore, the potential of Ba-Exo-induced ferroptosis inhibition in activating the Keap1-NRF2 pathway via p62 was investigated. The changes in Keap1 and NRF2 protein levels were evaluated using western blot, which showed increased NRF2 expression and decreased Keap1 expression after Exo and Ba-Exo treatment. Moreover, notable effects were seen after treatment with Ba-Exo (Figures 7(a)–7(c)). The immunofluorescence staining of NRF2 also confirmed that Ba-Exo treatment promoted the high expression of NRF2 protein (Figures 7(d) and 7(e)). Additionally, P62 downregulation in Ba-Exo attenuated the Keap1-NRF2 pathway activation (Figure 7(f)). Furthermore, NRF2 pathway inhibitor ML385 pretreatment was used to explore whether NRF2 pathway inhibition could attenuate the Ba-Exo-induced ferroptosis inhibition. Western blot results showed that ML385 significantly reversed the high expression of NRF2 protein caused by Ba-Exo (Figures 7(g) and 7(h)). Additionally, western blot of ferroptosis biomarkers indicated that the inhibition of the NRF2 pathway attenuated Ba-Exo-induced increase in GPX4 and SLC7A11 expressions and decrease in 5-LOX expression (Figures 7(g) and 7(h)). Meanwhile, ML385 significantly attenuated the Ba-Exo-induced high GSH and ROS contents and increased the intracellular Fe^2+^ and MDA levels (Figures 7(i)–7(m)). PI staining results also confirmed that ML385 reversed the increase in cell viability, which was promoted by Ba-Exos (Figure 7(n)). Therefore, the P62-Keap1-NRF2 pathway is involved in the ferroptosis inhibitory effect of Ba-Exo.

## 4. Discussion

The pathological manifestation of ALI includes a large number of liver cells that are severely damaged and undergo programmed necrosis, the inhibition of liver cell proliferation, severe damage of liver function, and failure of multiple organs in vivo [27]. Through in vivo and in vitro experiments, this study demonstrates for the first time that Ba-Exo can alleviate liver function damage and inhibit the occurrence of ferroptosis in hepatocytes to a greater extent than Exo. Moreover, P62 was significantly upregulated in Ba-Exo, whereas its downregulation in Ba-Exo counteracted the beneficial effect of Ba-Exo. P62 regulates hepatocyte ferroptosis by activating the Keap1-NRF2 pathway. The beneficial effect of Ba-Exo in inhibiting ferroptosis was also attenuated after the NRF2 pathway was inhibited. Therefore, Ba-Exo can be considered to be a promising therapeutic option for ALI. This study further elucidates the mechanism underlying the therapeutic effects of Ba-Exo.

The ability of exosomes to promote tissue regeneration and treat various diseases has been recently demonstrated. MSCs-Exo have been reported to contain cytokines, growth factors, mRNAs, miRNAs, and other biomolecules, which can regulate the metabolic activities of cells or tissues in vivo through the targeted delivery of these biomolecules [28]. Pretreated MSCs-Exo have been shown to improve therapeutic and transplantation efficacy. For example, melatonin-stimulated MSCs-Exo promotes diabetic wound healing by targeting the PTEN/AKT pathway to regulate macrophages M1 and M2 polarization [29]. Additionally, TNF-*α* pretreated MSCs-Exo modulate inflammation and osteoclastogenesis [30]. Several studies have shown that hypoxia-preconditioned MSCs alter miRNA content in their secreted exosomes, thereby enhancing their ability to promote cartilage repair in spinal cord injuries and osteoarthritis [31, 32]. Astragaloside has been shown to exert protective effects on liver function through multiple mechanisms. Shi et al. demonstrated that baicalin induces NRF2 cytoplasmic accumulation after APAP-induced ALI in mice, leading to NLRP3 inflammasome activation and subsequent increased IL-18 expression that induces hepatocyte proliferation, thereby promoting liver cell regeneration [24]. Furthermore, baicalin effectively prevents D-GalN/LPS-induced liver injury by inhibiting NF-kappaB activity and reducing TNF-*α* production, which can be attributed to HO-1 protein upregulation [33]. Additionally, Xu et al. report that baicalin confers a protective effect on zearalenone-induced liver injury by inhibiting the expression of oxidative stress, inflammatory cytokines, and caspase signaling pathways [34]. However, studies describing the use of Ba-Exo in ALI are scarce. In this study, TEM and NTA results showed that Ba-Exo and Exo had the same size, shape, and zeta potential. In vitro and in vivo studies also confirmed for the first time that Ba-Exo significantly reduced the elevation of ALT and AST and the release of pro-inflammatory cytokines after ALI compared to Exo, indicating its better protective effect on liver function.

Ferroptosis is a type of oxidative cell death induced by small molecular substances, which arise due to the imbalance between the generation and degradation of intracellular lipid ROS [2]. Cells synthesize GSH, which can eliminate free radicals, using glutamate cysteine synthase. Under normal physiological conditions, lipid peroxides are scavenged by GSH and GPX4, which reduces lipid peroxides to fatty alcohols or degrades them to hydroxy fatty acids. However, the inhibition of GSH and GPX4 synthesis promotes the peroxidation process and reduces the antioxidant capacity of cells [35]. Recently, ferroptosis has been associated with ALI, involving APAP-induced hepatotoxicity and D-GaIN/LPS-induced liver injury in mice [36, 37]. Ferroptosis plays an important role in these injuries, with various studies demonstrating that its inhibition attenuates ALI. Niu et al. demonstrated that protecting the mitochondria by inhibiting VDAC1 oligomerization attenuates ferroptosis in ALI [6]. Furthermore, ulinastatin attenuated ALI by inhibiting ferroptosis-related lipid peroxide accumulation, with the effects of UT1 mediated through the NRF2/HO-1 pathway and SIRT1 expression [38]. In this study, D-GaIN/LPS-induced liver injury increased iron content, lipid peroxides, and ROS. After Ba-Exo treatment, the increase in iron content, lipid peroxides, and ROS was significantly inhibited, indicating that Ba-Exo exerted a protective effect on liver function by inhibiting ferroptosis in hepatocytes. Subsequent in vitro experiments further confirmed that Ba-Exo was more effective than Exo in inhibiting the production of ROS and lipid peroxides caused by ferroptosis. Therefore, Ba-Exo attenuates iron accumulation in hepatocytes and exerts a protective effect on liver function.

Further experiments were performed to compare the inhibitory effect of Ba-Exo and Exo. It has been reported that specific proteins can be loaded into exosomes and delivered to target cells. For example, the exosome-mediated delivery of 14-3-3t to target cells modulates neuroinflammation and apoptosis, thereby exhibiting a protective effect in spinal cord injury [39]. Zhang et al. report that EGFR in exosomes secreted by gastric cancer cells can enter the liver and integrate into the plasma membrane of liver stromal cells to promote liver-specific metastasis [40]. Therefore, this study investigated the role of protein carriers in mediating the beneficial effects of Ba-Exo, wherein P62 was found to accumulate in Ba-Exo and exert ferroptosis inhibition and antioxidative stress effects in hepatocytes. Furthermore, the depletion of P62 in exosomes partially counteracted the beneficial effects of Ba-Exo. In addition to P62, Ba-Exo has the potential to carry various other proteins. Therefore, studies determining the role of various other proteins in ferroptosis inhibition are required.

The signaling pathway mediated by P62-Keap1-NRF2 is an important signaling pathway for maintaining oxidative stress and redox balance in the body [41]. Under nonstress conditions, low levels of NRF2 are mainly maintained by Keap1-mediated proteasomal degradation. However, under oxidative stress conditions, the conformation of the cysteine residue of Keap1 changes, dissociating the bound NRF2, which enters the nucleus and binds to ARE. This interaction regulates the expression of downstream antioxidant proteins and detoxification enzymes, thereby reducing oxidative stress damage to cells and tissues [42]. P62 is an important upstream regulator of the Keap1-NRF2 pathway. Under stress conditions, the expression of P62 protein is upregulated, which blocks the binding of Keap1 and NRF2 by competitively binding to Keap1 and inhibiting the ubiquitination of NRF2, thereby stimulating downstream gene expression [43]. Sun et al. demonstrated that the p62-Keap1-NRF2 antioxidant signaling pathway is a key negative regulator of iron toxicity in hepatocellular carcinoma cells via the transcriptional activation of genes involved in ROS and iron metabolism [26]. The inhibition of the P62-Keap1-NRF2 pathway can significantly enhance the anti-cancer activities of Erastin and Sorafenib in liver cancer cells in vivo and in vitro [26]. This study shows that Ba-Exo significantly upregulates P62 protein content in target cells, inhibits NRF2 protein degradation, and reduces Keap1 protein expression via P62, thereby exerting antioxidative stress effect and inhibiting ferroptosis.

## 5. Conclusions

Ba-Exo exerts a protective effect on liver function and activates the Keap1-NRF2 pathway via P62, thereby inhibiting ROS production and lipid peroxide-induced ferroptosis. Therefore, baicalin pretreatment is an effective and promising approach in optimizing the therapeutic efficacy of Exo in ALI.

## Data Availability

The datasets used and analyzed during the current study are available from the corresponding authors on reasonable request.

## Abbreviations

D-GaIN/LPS: D-galactosamine and lipopolysaccharide
ALI: Acute liver injury
MSCs: Mesenchymal stem cells
BMSCs: Bone marrow mesenchymal stem cells
MSCs-Exo: MSCs-derived exosomes
Ba-Exo: Exosomes pretreated with baicalin
DMEM: Dulbecco's modified Eagle's medium
TEM: Transmission electron microscopy
NTA: Nanoparticle tracking analysis
ALT: Alanine aminotransferase
AST: Aspartate aminotransferase
LPS: Lipopolysaccharide
ROS: Reactive oxygen species.
